# Mouth-Breathing

**Published:** 1886-12

**Authors:** 


MOUTH-BREATHING.—"The New York *World* prints an interview with a well-known physician on the pernicious mouth-breathing habit, "Why," said the doctor," you can tell one of those mouth-breathers anywhere the moment you see him. From disease of the nose his lips are retracted, his mouth is continually open, his gums recede and his teeth protrude, particularly those in the upper jaw; the flesh that forms the lower part of the nostrils is shrunken, the openings of the nostrils are diminished in size, there are wrinkles at the outer edges of the eyes, and deep lines run from the nostrils to the angles of the mouth. These all give the person an expression of either idiocy, silliness, or suffering."

It is said that a man can inhale mephitic air through the nose for a certain time in the bottom of a well without harm, but if he opens his mouth to answer a question or call for help, his lungs are closed and he expires."

If such are the effects of mouth breathing, it would be best to choose the least of two evils and keep the mouth full of tobacco juice.—ED.

